# Imprinted Oxide and MIP/Oxide Hybrid Nanomaterials for Chemical Sensors †

**DOI:** 10.3390/nano8040257

**Published:** 2018-04-20

**Authors:** Adeel Afzal, Franz L. Dickert

**Affiliations:** 1 Department of Chemistry, College of Science, University of Hafr Al Batin, P.O. Box 1803, Hafr Al Batin 31991, Saudi Arabia; aa@aafzal.com; 2 Department of Analytical Chemistry, University of Vienna, Währingerstraße 38, 1090 Vienna, Austria

**Keywords:** chemical sensors, complex mixtures, assay, hybrid nanomaterials, metal oxides, molecular recognition, molecularly imprinted polymers

## Abstract

The oxides of transition, post-transition and rare-earth metals have a long history of robust and fast responsive recognition elements for electronic, optical, and gravimetric devices. A wide range of applications successfully utilized pristine or doped metal oxides and polymer-oxide hybrids as nanostructured recognition elements for the detection of biologically relevant molecules, harmful organic substances, and drugs as well as for the investigative process control applications. An overview of the selected recognition applications of molecularly imprinted sol-gel phases, metal oxides and hybrid nanomaterials composed of molecularly imprinted polymers (MIP) and metal oxides is presented herein. The formation and fabrication processes for imprinted sol-gel layers, metal oxides, MIP-coated oxide nanoparticles and other MIP/oxide nanohybrids are discussed along with their applications in monitoring bioorganic analytes and processes. The sensor characteristics such as dynamic detection range and limit of detection are compared as the performance criterion and the miniaturization and commercialization possibilities are critically discussed.

## 1. Introduction

Metal oxides are high density semiconducting solids that possess excellent electrochemical and sensing properties [1]. Since 1962, the oxides of various metals have been used as the key recognition elements in chemical and gas sensors [2,3,4,5]. The advantages of using metal oxides as sensors include their cost-effectiveness, high chemical and thermal stability, excellent mechanical and electrical properties, and their compatibility with different electronic, optical, and gravimetric or acoustic devices [6]. In addition, the advent of nanotechnology and continuous research have led to the development of processes and procedures for easy fabrication of different metal oxide nanostructures [7,8,9,10]. Today, most metal oxides can effortlessly be prepared from inexpensive metal salt or metal alkoxide precursors using facile, one-step chemical or physical approaches [11,12,13,14,15,16,17]. These nano-structured metal oxides are extremely sensitive to minor changes in the chemical environment atop their surface owing to their high aspect ratio. However, metal oxides have poor specificity when it comes to sensing different molecular targets in complex mixtures or real samples.

Molecular imprinting [18,19,20,21], on the other hand, is an innovative approach to induce specificity and enhance selectivity toward any target. Molecularly imprinted polymers, often denoted as molecularly imprinted polymers (MIP), are the most commonly used antibody mimics for selective recognition of different analytes ranging from small organic compounds to biologically relevant macromolecules, cells and microorganisms [22,23,24,25]. Albeit, molecularly imprinted metal oxides are less common partly because of better efficacy of MIP and partly due to the rigid nature and intrinsic hardness of oxides that is indeed encouraging in terms of their greater stability and applicability in harsh environments, but somehow compromises their sensitivity compared to MIP. Nonetheless, surface and bulk imprinting of metal oxide sol-gel phases and nanomaterials is considered a great tool to improve the material’s specificity and its applicability in complex mixtures [26,27,28,29]. Therefore, molecularly imprinted sol-gel phases and metal oxide nanoparticles have attracted numerous researchers and scientists to produce highly specific and selective materials for molecular recognition and sensing applications [30,31,32,33,34,35,36,37].

In addition to imprinted sol-gel and metal oxide nanoparticles, the tendency to incorporate metal oxides in MIP to construct MIP/oxide hybrid coatings and to design multicomponent MIP/oxide containing hybrids has substantially grown over the past few years to achieve superior sensor performance. In this article, we present an overview of different design strategies for the synthesis of molecularly imprinted sol-gel phases and metal oxide nanoparticles, MIP/oxide nanohybrids, and multicomponent hybrids or composites containing MIP and oxide-based nanomaterials as the selective recognition elements for electrochemical, optical, and gravimetric transducers. Subsequently, we discuss the applications of these imprinted oxide and MIP/oxide hybrid materials in the selective detection of biologically relevant molecules, harmful organic substances, drugs, and other molecular or cellular targets in complex mixtures. Finally, the performance of various chemical sensors is critically analyzed, and future research directions are discussed with special emphasis on specificity and stability of imprinted oxide and MIP/oxide hybrid-based sensors.

## 2. Synthesis and Fabrication of Recognition Elements

In an effort to develop chemical sensors that can specifically respond to a targeted compound, the scientists around the globe have proposed a number of innovative strategies involving synthesis and fabrication of selective recognition elements based on molecularly imprinted oxides and MIP/metal oxide nanohybrids. The following sections highlight the state-of-the-art approaches to construct highly selective sensors for various analytical applications.

### 2.1. Formation of Molecularly Imprinted Sol-Gel Materials

The sol-gel process is a straightforward method to produce highly robust and scratch-resistant coatings on any surface [38,39]. However, a simple sol-gel material is barely selective to different analytes of interest. The molecularly imprinted and appropriately functionalized sol-gel materials are sensitive and selective [40,41,42]. For instance, Latif et al. [43] have produced titanate sol-gel coatings imprinted with various aliphatic hydrocarbons by TiCl_4_ initiated hydrolysis of different Ti-alkoxide precursors in *iso*-propanol solution containing 0.1% water. The sensitivity of these hydrocarbon imprinted sol-gel coatings is optimized by changing Ti-alkoxide precursors and the resulting sensor is found to differentiate between structural isomers, *n*-octane and *iso*-octane. The selective SiO_2_ and TiO_2_ sol-gel coatings for mass-sensitive detection of oxidized engine oil products are also prepared in a similar sol-gel process using capric acid as the template [44]. In this case, the sensor performance is optimized by choosing appropriate functional monomer such as aminopropyltriethoxysilane (APTES). These imprinted sol-gel layers can be easily fabricated on electronic or acoustic devices by spin-coating sol solution [43,44].

**Figure 1:**
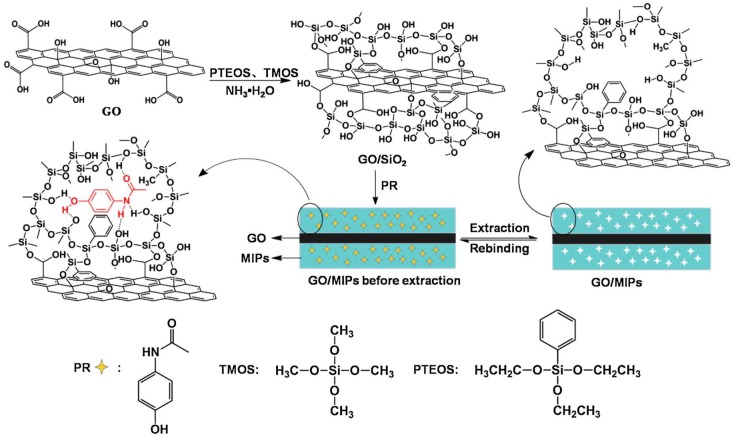
A schematic for preparing paracetamol imprinted sol-gel on graphene oxide surface. Reproduced from [50] with permission from Springer, 2014.

Earlier, it has been demonstrated that pristine sol-gel coatings on the surface of a transducer can be imprinted with living yeast cells using a soft-lithographic surface stamping method [26]. This leads to rugged surface patterns specific to the dimensions of yeast cells. Furthermore, there are many reports on synthesis and fabrication of hybrid sol-gel materials as recognition elements for various applications [45,46,47,48,49]. Luo et al. [50] have synthesized a paracetamol imprinted sol-gel layer on graphene oxide (GO) via base catalyzed hydrolysis of tetramethylorthosilicate (TMOS) and phenyltriethoxysilane (PTEOS). Figure 1 shows a schematic of the synthesis of imprinted sol-gel/GO hybrid material. In a similar sol-gel process, Zhou et al. [51] have prepared a tetracycline imprinted SiO_2_ sol-gel layer on the surface of ZnO nanorods, which improves sensitivity and imparts selectivity to the sensor.

### 2.2. Synthesis of Molecularly Imprinted Metal Oxide Nanoparticles

**Figure 2:**
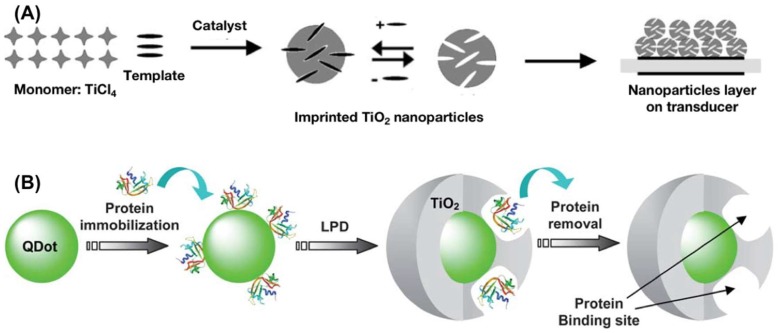
(**A**) A schematic for synthesis of molecularly imprinted TiO_2_ nanoparticles. Adapted from [28] with permission from Springer, 2007; (**B**) A schematic illustration of protein imprinted TiO_2_ coated quantum dots. Reproduced from [57] with permission from Royal Society of Chemistry, 2011.

Synthesis of molecularly imprinted metal oxide nanoparticle is a step forward in fabricating more responsive chemical sensors due to the higher surface area availability for analyte’s interaction with the receptor materials [52]. The imprinted metal oxide nanoparticles have therefore found several applications in molecular recognition and catalysis [53,54,55,56]. Lieberzeit et al. [28] have tested capric acid imprinted TiO_2_ nanoparticles for the detection of oil degradation products in used automotive oils. The imprinted TiO_2_ nanoparticles can be prepared by simple hydrolytic processing of TiCl_4_ in the presence of template molecules and a dilute aqueous ammonia as catalyst, as shown in Figure 2A. The imprinted TiO_2_ nanoparticles show high sensitivity and great applicability in complex mixtures.

Inoue et al. [57] have prepared protein imprinted TiO_2_ coated quantum dots (QD) by a liquid phase deposition (LPD) procedure. LPD solution is prepared by dissolving the precursor ammonium hexafluorotitanate and boric acid and pH of the solution is adjusted to 7 by aqueous ammonia. Carboxylated QD, LPD solution, bovine pancreas ribonuclease A (BPRNase; template protein), and the covalent coupling agent 1-ethyl-3-(3-dimethylaminopropyl) carbodiimide hydrochloride (EDC.HCl) are then mixed in a phosphate buffer and are reacted to yield imprinted TiO_2_/QD hybrids. Figure 2B illustrates the procedure for synthesis of imprinted TiO_2_/QD. These fluorescent, imprinted TiO_2_/QD can selectively detect and capture BPRNase. Recently, Gao et al. [58] have utilized an innovative approach to prepare magnetic, fluorescent nanoparticle core with a ciprofloxacin imprinted silica shell. Oleic acid capped Fe_3_O_4_ nanoparticles and CdTe QD are mixed with APTES and TEOS and the hydrolysis is carried out by aqueous ammonia in the presence of template and cetyltrimethylammonium bromide (CTAB). The strategies mentioned above are a few of the selected examples of imprinted oxide nanoparticles used as recognition elements in acoustic and fluorescent sensors.

### 2.3. Fabrication of MIP/Metal Oxide Hybrid Nanostructures

A number of different strategies for fabrication of MIP/metal oxide hybrid nanostructures have been proposed so far [59,60,61,62]. In principle, these strategies can be divided into two sub-categories depending on the type of MIP/oxide adduct or hybrid produced: (a) the processes combining metal oxides and MIP in the presence or absence of a covalent crosslinker to prepare MIP/oxide bulk type nanohybrids [63,64]; and (b) the processes focusing on the fabrication of MIP on the surface of metal oxide nanostructures leading to the formation oxide/MIP core-shell type hybrid nanostructures [65,66]. For example, Patra et al. [67] have fabricated functional ZnO nanoparticles on vinyltriethoxysilane modified graphite pencil electrode (GPE) and polymerized a calcitonin imprinted film on their surface to obtain the bulk MIP/ZnO hybrid that contained ZnO nanoparticles dispersed within the MIP matrix.

**Figure 3:**
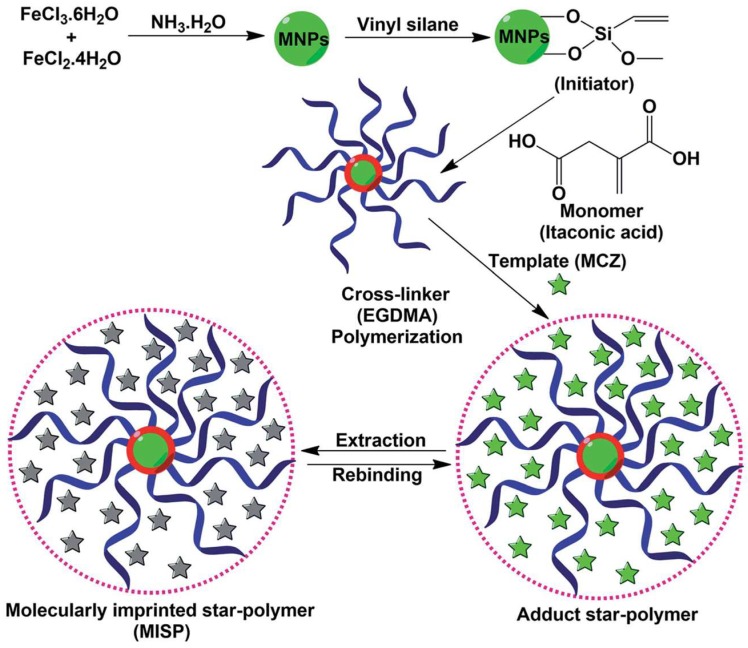
Graphical representation showing fabrication of mancozeb imprinted star polymer on the surface of Fe_3_O_4_ nanoparticles. Reproduced from [69] with permission from Royal Society of Chemistry, 2016.

On the other hand, Wang et al. [68] have synthesized thionine imprinted polydopamine on the surface of magnetic Fe_3_O_4_ nanoparticles to develop Fe_3_O_4_-core MIP-shell type nanohybrid. Kumar et al. [69] have also designed an oxide/MIP core-shell type nanohybrid having a superparamagnetic Fe_3_O_4_ nanoparticle core and mancozeb imprinted star polymer shell. Figure 3 illustrates the synthesis of Fe_3_O_4_/MIP core-shell type hybrids used for the detection of a pesticide. In this process, Fe_3_O_4_ nanoparticles are first prepared and surface functionalized with a vinyltriethoxysilane monomer. Functionalized Fe_3_O_4_ nanoparticles are then reacted with ethylene glycol dimethacrylate (EGDMA) in the presence of a template to yield an imprinted star polymer on the surface of Fe_3_O_4_ nanoparticles, as shown in Figure 3. In summary, both these strategies lead to the fabrication of highly sensitive and selective MIP/oxide hybrid recognition elements, which indeed benefit from the synergistic properties of MIP and oxide nanoparticles.

### 2.4. Fabrication of Multicomponent Hybrids Containing MIP and Metal Oxides

**Figure 4:**
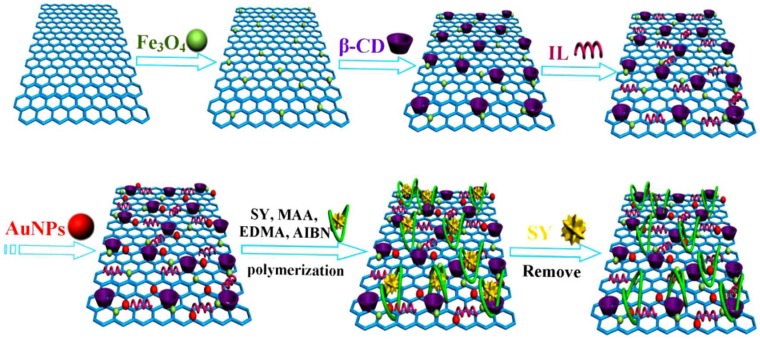
Schematic diagram of synthesis of a multicomponent molecularly imprinted hybrid material (molecularly imprinted polymers (MIP)/Fe_3_O_4_/GO/β-CD/IL/Au). Reproduced from [74] with permission from Elsevier, 2016.

In many cases, the recognition elements consist of more than two sensitive components to improve the ultimate sensor performance [70]. In such multicomponent hybrid materials, every constituent has discrete properties and contributes differently toward the sensitivity and selectivity of the device. To achieve the best sensor performance and applicability in real samples, several efforts have been focused on fabricating composite materials containing MIP, metal oxides, and other nanomaterials [70,71,72,73]. Li et al. [74], for instance, have designed a multicomponent hybrid material containing MIP, Fe_3_O_4_, and GO. They have first synthesized GO followed by in situ deposition of Fe_3_O_4_ nanoparticles to prepare magnetic GO. β-Cyclodextrin (β-CD), ionic liquid (IL), and gold (Au) nanoparticles are subsequently deposited in a step-wise process. Finally, MIP is synthesized in situ using precipitation polymerization of methacrylic acid in the presence of sunset yellow (SY) template to yield MIP/Fe_3_O_4_/GO/β-CD/IL/Au hybrid material. Figure 4 illustrates the step-by-step fabrication of a multicomponent hybrid material.

## 3. Applications

### 3.1. Electrochemical Sensing of Biologically Relevant Molecules

A number of biologically significant compounds and biomacromolecules have been selectively detected using metal oxides and MIP hybrids. These include compounds bioavailable from fruits, vegetables, and other food products as well as those produced by the human body such as neurotransmitters, amino acids, proteins, and various disease biomarkers. Table 1 provides an overview of the sensing characteristics of MIP/oxide nanohybrids for the electrochemical detection of some biologically relevant molecules.

A phenolic compound, *trans-*resveratrol or 3,5,4′-trihydroxy-*trans*-stilbene, is produced by certain plants to combat injury and fungal infections [75]. It is a phytoalexin present in peanuts, grapes, and wines in high concentrations and has strong anti-inflammatory and antioncogenic properties [76]. Its presence in food and wine means that it can be detected in human blood stream [77]. Xiang and Li [78] have developed an electrochemical sensor based on indium tin oxide electrodes modified by γ-methacryloxypropyltrimethoxysilane and resveratrol imprinted polymer film. The sensor is capable of detecting very low concentrations (0.8 μM) of resveratrol in phosphate-buffered saline (PBS) solution. The sensor also shows good selectivity toward *trans-*resveratrol compared to the selected template analogs bisphenol-A and polydatin.

A pentacyclic triterpenoid, oleanolic acid, is another bio-relevant molecule that is present in many food products and human serum [84]. It has been known for its antihypertensive, antidiabetic, and anticancer effects [85,86,87,88]. Zhang et al. [80] have fabricated an electrochemical sensor by modifying multi-walled carbon nanotubes (MWNT) with SnO_2_ nanoparticles and molecularly imprinted polypyrrole. The imprinted sensor exhibits excellent sensitivity and selectivity toward oleanolic acid compared to ursolic acid. The sensor is successfully used to determine the concentration of oleanolic acid in *Acitinidia deliciosa* root samples that is found to be 15.65 μg/L.

Dopamine, a catecholamine neurotransmitter, is one of the most significant compounds in human central nervous system and its deficiency is linked to emotional and neurological disorders, e.g., Parkinson’s disease [89,90]. Therefore, in vivo and in vitro detection of dopamine is extremely important for clinical and medical applications [91]. In recent times, many researchers have tried to develop receptors for selective recognition of dopamine [92,93,94]. For instance, Wang et al. [65] have developed MIP arrays supported on ZnO nanotubes as the recognition elements for electrochemical detection of dopamine. The sensor exhibits two dynamic linear detection ranges: 0.02–5 μM and 10–800 μM. The proposed sensor is used for the determination of dopamine in more complex biological samples, i.e., human urine, and shows good accuracy.

**Figure 5:**
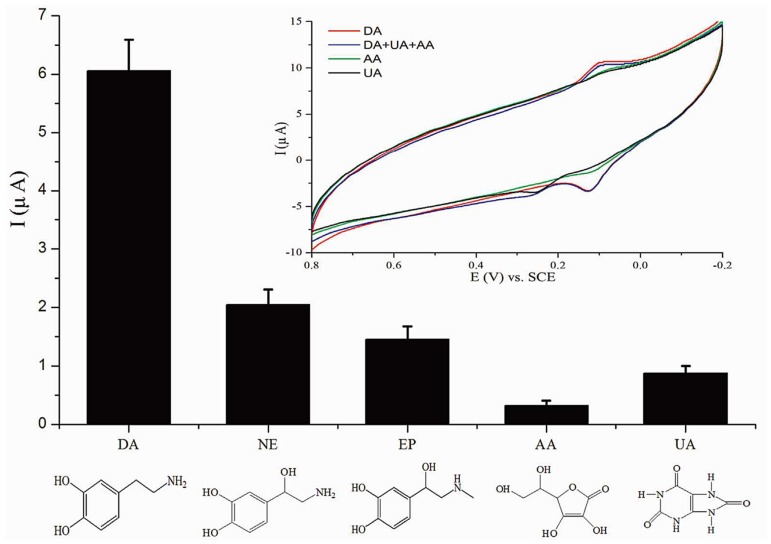
Current responses of an electrochemical sensor made of MIPs/CuO@GCE electrode toward dopamine (DA), norepinephrine (NE), epinephrine (EP), ascorbic acid (AA), and uric acid (UA) and the structural formulas of five compounds. The concentration of each compound is 25 μM. Inset: The cyclic voltammograms of 2 μM DA in the absence and presence of 50 μM UA and 50 μM AA. The concentrations are equal to the upper limit of normal reference in serum. Reproduced from [63] with permission from Elsevier, 2015.

Earlier, Li et al. [63] have demonstrated the use of imprinted poly(nicotinamide)/CuO nanoparticles hybrid as the sensitive element for dopamine determination. The hybrid sensor shows high sensitivity for dopamine with a detection limit of 8 nM and minimal interference with its structural analogs: uric acid, ascorbic acid, epinephrine, and norepinephrine (as shown in Figure 5). The sensor is also used to determine dopamine content in human serum samples showing excellent recoveries. Zeng and coworkers [81] have also studied dopamine concentrations in human urine samples using an electrochemical sensor composed of SiO_2_-coated graphene oxide and MIP composite. The sensor exhibits 3.2 times higher sensitivity toward dopamine compared to a non-imprinted sensor, wide linear detection range (0.05–160 μM), and negligible interference from norepinephrine and epinephrine.

Norepinephrine is also a catecholamine neurotransmitter and a hormone that is present in the mammalian central nervous system and performs various physiological functions [95,96,97]. A recent study demonstrates that MIP and Sb-doped SnO_2_-SiO_2_ composite sol coated single-walled carbon nanotubes (SWNT) make a highly efficient hybrid electrochemical sensor for norepinephrine [82]. The sensor is successfully applied to determine norepinephrine concentrations in human blood serum samples showing 99.67% recovery. The sensor also displays nanomolar (33.3 nM) limit of detection that is either comparable or significantly better than other reported sensors, e.g., based on Fe_3_O_4_-doped phthalocyanine/MWNT [98], synthesized hydroquinone/carbon nanotubes (CNT) [99], synthesized acrylic acid/ZnO nanorods [100], and ionic liquid/CNT nanocomposite [101].

Amino acids are relatively smaller biomolecules that are particularly relevant and are essentially present in our bodies [102]. They are the fundamental constituents of proteins and also participate in the biosynthesis of catecholamines discussed above [103]. It is important to monitor their concentrations in human serum, urine, saliva, etc. owing to their significance and functions. For example, an amino acid tyrosine is considered to be an important disease biomarker for lung cancer and acute ischemic stroke [104,105]. Saumya et al. [64] have designed and organic-inorganic hybrid electrochemical sensor for tyrosine detection in urine samples. The sensor is based on molecularly imprinted polypyrrole and CuO nanohybrid and exhibits very low detection limit and appreciable selectivity toward tyrosine, albeit it is unable to differentiate between the enantiomers (l- and d-tyrosine). Nonetheless, tyrosine detection is attributed to the copper-tyrosine phosphate complexation and electrocatalytic mechanisms [106].

Another important cancer biomarker for oral cancer diagnosis is the dimeric protein, interleukin-8, that is overexpressed in human during inflammations and head, neck, and pancreatic cancers [107,108]. Its successful, non-invasive recognition can help early diagnosis of cancers. Recently, Tang et al. [83] have fabricated an electrochemical sensor by interleukin-8 surface imprinting polymer on graphene oxide modified Fe_3_O_4_ nanoparticles. The sensor shows very low detection limit (0.04 pM) and good resistance to non-specific protein binding. The sensor can determine interleukin-8 in saliva samples. Calcitonin, a polypeptide hormone produced by C-cells of thyroid gland, is a clinical biomarker for migraine and medullary thyroid carcinoma [109,110]. Patra et al. [67] have employed a surface imprinting procedure to prepare calcitonin imprinted polymer on ZnO nanostructures by atom transfer radical polymerization on vinyl-modified electrode surface. The electrochemical sensor formed as a result displays low detection limit (3.09 ng/L), excellent selectivity, and remarkable applicability in human blood samples showing approximately 100% recovery.

### 3.2. Sensing Harmful Organic Compounds

A number of functional organic molecules are frequently utilized in industry as building blocks for polymeric and plastic products; in agriculture as pesticides and insecticides; and in drinks and food products as colors and flavors [111]. The excessive use of these compounds leads to their omnipresence in fruits, vegetables, soil, and water [111,112]. Some of these organic compounds are extremely harmful for living species including human beings and raise major concern for their detection, for instance, in drinking water and edible products. How imprinted oxide nanoparticles and MIP/oxide based nanohybrids have been employed in monitoring these hazardous chemicals is discussed in this section. Table 2 provides a brief account of various sensor systems and their performance characteristics in detecting the selected organic compounds.

Bisphenol A [123,124] is widely used in industry for the preparation of epoxy resins and polycarbonate plastics, which in turn are used in the production of sealants, coatings, varnishes, insulations, packaging, bottles, medical apparatus, eyeglass lenses, etc. It has been linked to numerous health effects including female infertility and carcinogenicity [125,126,127]. Qiu et al. [113] have first fabricated a fluorescence sensor based on SiO_2_-coated CdTe QD having the MIP shell for selective determination of bisphenol A. The sensor is tested in river water and milk samples and demonstrates 96.31% recovery with low relative standard deviation (RSD). Zhang et al. [114] have also tested a photoelectrochemical sensor in bisphenol A spiked tap water and river water showing 89% and 96% recoveries, respectively. The sensor is composed of MIP-coated SnO_2_ nanoparticles assembled on ITO electrode, and shows low detection limit (1.2 nM) and good selectivity toward bisphenol A.

**Figure 6:**
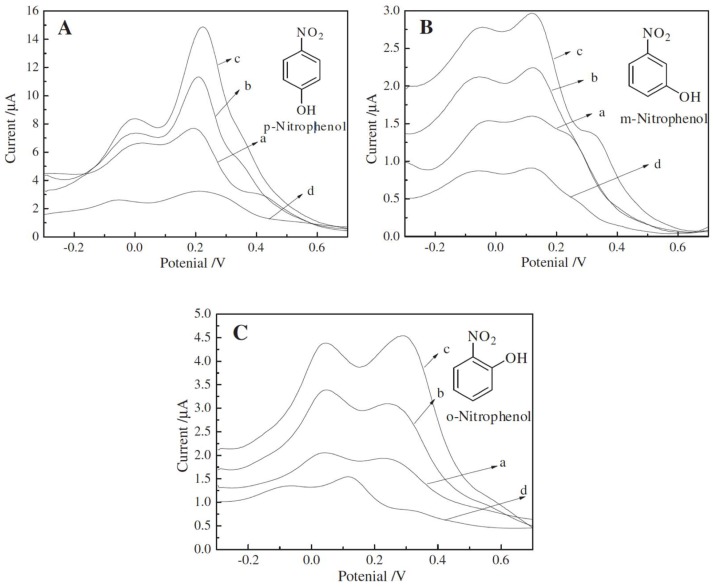
Square wave voltammograms (SWV) of the electrochemical sensor composed of MIP/ZnO-MWNT-CS@ITO electrodes with different concentrations (a) 1.0 × 10^−6^ M; (b) 1.0 × 10^−5^ M; (c) 1.0 × 10^−4^ M, and (d) NIP/ZnO-MWNT-CS@ITO with 1.0 × 10^−4^ M concentration of (**A**) *p*-nitrophenol; (**B**) *m*-nitrophenol; and (**C**) *o*-nitrophenol in PBS solutions. Reproduced from [117] with permission from Elsevier, 2012.

4-Nonylphenol is the potential endocrine disrupting chemical and is ubiquitously present in drinking water and edible products [128]. An accurate and fast 4-nonylphenol detection method using a multicomponent MIP/Au/TiO_2_ nanocomposite based electrochemical sensor is reported [116]. The nanocomposite sensor is capable of detecting trace levels of 4-nonylphenol in tap water and pomfret samples with 95% recovery. *p-*Nitrophenol is another phenolic compound that is widely used in the production of dyes and drugs and has toxic anthropogenic effects [129,130,131]. Hu et al. [117] have designed an electrochemical sensor by coating an imprinted sol solution on top of ZnO-MWNT-CS hybrid modified ITO electrode. The imprinted sensor exhibits very low detection limit (1 nM) and is able to determine *p*-nitrophenol concentration in spiked water samples. The square wave voltammograms (SWV) of MIP/ZnO-MWNT-CS@ITO sensor reveal notable selectivity toward *p*-nitrophenol compared to *o*-nitrophenol and *m*-nitrophenol (see Figure 6).

17β-Estradiol, a natural manure-borne hormone that stimulates and regulates the growth and conservation of female genotype in mammals, is often detected in surface water close to the agricultural fields or water treatment facilities [132]. It has been designated as one of the endocrine disrupting chemicals by the US Environmental Protection Agency [133,134]. 17β-Estradiol, even at low concentrations (ng/L), can negatively affect mammals by disrupting endocrine function and altering secondary sex characteristics, which may result in reproductive system diseases [134,135,136]. Li et al. [118] have developed a highly sensitive electrochemical sensor for detecting 17β-Estradiol in water that is based on MIP coated Fe_3_O_4_ nanobeads immobilized on reduced graphene oxide (Fe_3_O_4_-MIP/rGO). The electrochemical sensor displays excellent selectivity and is able to detect trace levels (<5 μM) of 17β-Estradiol in water. Fe_3_O_4_-MIP@rGO sensor also demonstrates low detection limit compared to previously reported [137] electrochemical sensors based on MIP-coated Pt nanoparticles immobilized on rGO (MIP-Pt/rGO).

Amaranth is a typical organic azo-dye that has been detected in soft drinks and sweets using various electrochemical sensors [138,139,140]. However, excessively high concentrations of amaranth may have adverse cytotoxic and genotoxic effects [141,142]. Han et al. [119] have designed an electrochemical sensor by fabricating Fe_3_O_4_@rGO-doped MIP membrane on magnetic glassy carbon electrodes (GCE). The sensor exhibits good reproducibility and stability, adequate selectivity, and moderately low limit of detection. The sensor is tested in various fruit drinks containing grapes, watermelon, and peach flavors and demonstrates 93–100% recovery. Duan et al. [120] have developed a chemiluminescence method for the selective detection of vanillin, another widely used flavoring substance in foods and beverages. The compound is regarded as harmful at high concentration [143]. The chemiluminescence sensor is based on a multicomponent hybrid made of MIP/Fe_3_O_4_-GO nanostructures and is shown to determine vanillin in real samples such as vanilla slice, vanilla milk tea, and vanilla drink [120]. It also shows low interference with vanillin’s structural analogs and lower detection limit compared to many reported electrochemical systems due to the imprinting effect [144,145,146].

Pesticides and insecticides are a group of harmful organic compounds that are widely spread in soil and water, and often accumulate in agricultural food products [147,148]. There are many examples of analytical methods and devices for recognition of pesticides and insecticides in soil, water, and food samples [149,150]. Kumar et al. [69] have demonstrated the successful determination of mancozeb, a general use pesticide, in some soil and water samples with 95–100% recovery. They have employed MIP-modified superparamagnetic Fe_3_O_4_ nanoparticles to achieve high sensitivity and selectivity for the target analyte. Recently, Sun et al. [121] have fabricated a photoelectrochemical sensor based on MIP-modified hierarchically branched TiO_2_ nanorods. The sensor offers a promising platform for highly sensitive detection of chlorpyrifos with limit of detection as low as 7.4 pg/mL. The proposed method also exhibits chlorpyrifos sensitivities comparable to highly sophisticated GCMS assay in spiked water samples.

### 3.3. Monitoring Drug Concentrations

Drugs are synthetic or naturally occurring organic compounds that are active against various diseases. It is important to measure their concentration in human blood serum to administer drug dosage [151,152,153]. Imprinted oxides and MIP/oxide nanohybrids have also found applications in drug assay and therapeutic drug monitoring. They have shown substantial promise in this regard. Table 3 shows the selected examples of fluorescence and electrochemical sensor systems used to monitor different drugs in human serum, urine, or spiked samples.

Tetracycline, a broad-spectrum antibiotic for bacterial infections, is among the most studied antibacterial agent for therapeutic drug monitoring [159,160,161,162]. Zhou et al. [51] have designed a fluorescent sensor based on mesoporous MIP sol-gel grown on the surface of ZnO nanorods for the selective recognition of tetracycline. The mesoporous sensor shows high sensitivity with imprinting factor equal to 3.50 and considerable selectivity compared to related drugs, e.g., oxytetracycline and doxycycline. The mesoporous sensor also reveals excellent recoveries (>100%), when tested in water samples spiked with 6–24 μM tetracycline concentrations. The sensor without mesoporous structure does not possess appreciable drug sensing properties.

Wang et al. [155] have utilized a similar method in developing the first electrochemical sensor for the detection of procainamide hydrochloride, a drug used for the treatment of cardiac arrhythmias. The sensor is based on a molecularly imprinted oligomeric methyl silsesquioxane (MSSQ) and TiO_2_ composite sol coated GCE. It exhibits good molecular recognition properties with 96.77–101.35% recoveries, when applied to monitor procainamide concentrations in human blood serum. It is also noticed that the inclusion of TiO_2_ in MSSQ sol significantly enhances the sensitivity.

**Figure 7:**
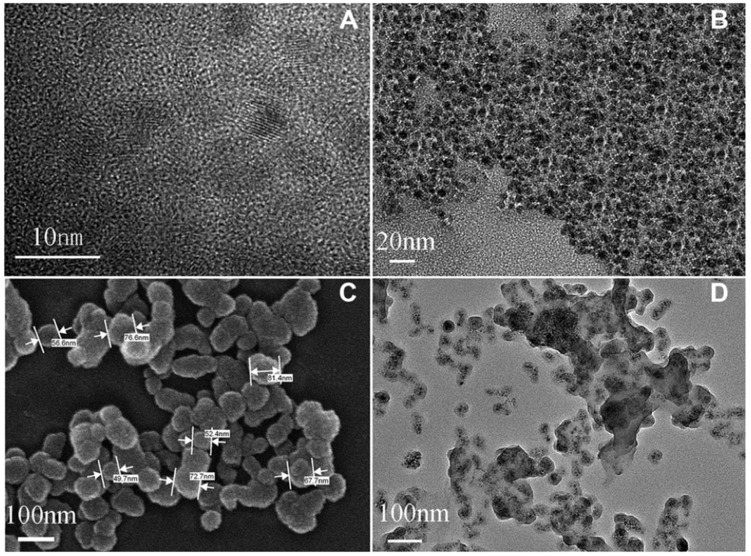

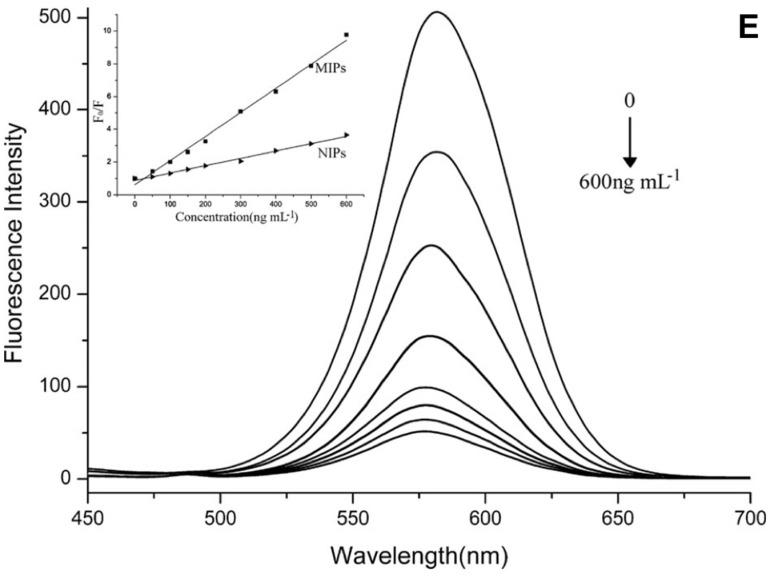
(**A**) HR-TEM images of CdTe QD; (**B**) TEM image of oleic acid capped Fe_3_O_4_ nanoparticles; (**C**) SEM image and (**D**) TEM image of MIP; (**E**) Fluorescence spectra of the MIPs aqueous solution with the increasing concentrations of ciprofloxacin. Inset: Stern-Volmer-type description of the data showing a linear fit throughout the ciprofloxacin concentration range. Adapted from [58] with permission from Wiley, 2014.

Ciprofloxacin is also used to treat bacterial infections and many scientists have been developing a reliable tool to determine its concentration in real samples [163,164,165]. Gao et al. [58] have applied DFT studies and computational simulation to design a fluorescent sensor based on Fe_3_O_4_-CdTe core and imprinted SiO_2_ shell formed by a sol-gel process. Figure 7A–D shows the microstructure and surface morphology of different recognition elements and Figure 7E illustrates the fluorescence spectra of the recognition element with increasing concentrations of ciprofloxacin. It is observed that the fluorescent sensor is suitable for the selective recognition of ciprofloxacin in spiked human urine samples. The sensor also presents superior sensitivity and lower detection limit compared to MIP-based micromechanical cantilever sensor system [166].

Luo et al. [50] have presented a one-pot synthesis of GO/imprinted-SiO_2_ sol-gel hybrid material to fabricate electrochemical sensor platform for paracetamol detection. This GO/MIP sol-gel sensor displays good recognition ability toward paracetamol compared to its analogs, excellent stability, and is successfully applied to determine drug concentration in tablets and spiked urine samples. Compared to a layer-by-layer assembled polymer/MWNT composite-based electrochemical sensor [167], GO/MIP sol-gel sensor exhibits broader linear response range and lower detection limit for paracetamol. Lei et al. [156] have fabricated an ATO-doped MIP sol-gel on Pt electrodes for the detection of various β_2_-agonists. β_2_-Agonists such as clenbuterol are synthetic bronchodilators used to treat asthma [168]. The ATO/MIP sol-gel sensor shows template-specific β_2_-agonists recognition ability and resistance to structurally analogous coexisting molecules. Moreover, the proposed sensor is satisfactorily applied to determine β_2_-agonists in human serum samples with recoveries greater than 94.6%.

Luteolin, a flavone present in many plants, is a potential drug for inflammations and cancer prevention and therapy [169,170,171]. In the last few years, several efforts have been focused on constructing highly sensitive electrochemical devices for luteolin detection in spiked samples as well as in real food products [172,173,174,175]. Xu et al. [158] have also fabricated an electrochemical sensor using imprinted poly-carbazole electropolymerized on MoS_2_/GN-CNT nanocomposite coated GCE. The imprinted sensor displays excellent sensitivity (381.3 μA/μM·cm^2^) and selectivity toward luteolin compared to tens of other chemicals, ions, and structural analogs. The sensor is successfully applied to determine luteolin concentrations in food samples and the luteolin content measured in carrots and chrysanthemum tea are 0.21 and 3.9 mg/g, respectively. These results also show good agreement with the values determined by more sophisticated HPLC analysis.

### 3.4. Monitoring Real-Time Processes with Gravimetric Sensors

The gravimetric or mass-sensitive sensors are highly efficient piezoelectric devices capable of precisely detecting any analyte in the gas/liquid phases [176,177]. The piezoelectric devices such as quartz crystal microbalance (QCM), surface acoustic wave (SAW), and surface transverse wave (STW) resonators are often combined with MIP and/or oxide-based materials to produce highly selective chemical sensors for a variety of different analytes [178,179]. Herein, we present an overview of the imprinted sol-gel or metal oxide nanoparticle based gravimetric sensors used to monitor and control real-time processes, e.g., oxidative degradation of engine oil. The automotive engine oil is a complex mixture of hydrocarbons and additives, and is used as lubricant to reduce friction, dissipate thermal energy, and avoid or delay corrosion in an internal combustion engine [180]. The oxidative degradation of oil during regular engine operation diminishes its functions and may lead to wearing of components [181,182]. Thus, it is important to monitor real-time oxidative degradation processes and oil quality during the engine operation. A number of analytical tools for on-board assessment of oil quality have been designed and tested in the past [183].

**Figure 8:**
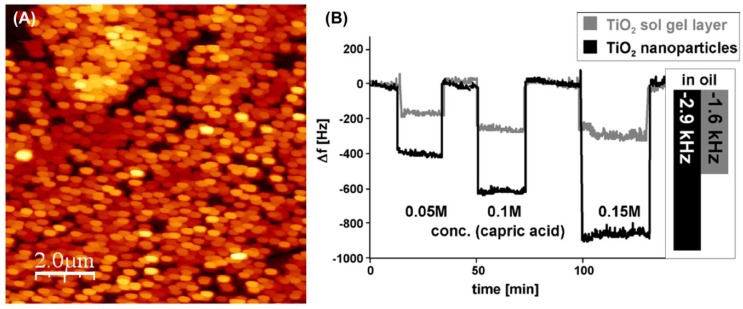
(**A**) AFM image of molecularly imprinted TiO_2_ nanoparticles fabricated on a transducer surface; (**B**) Quartz crystal microbalance (QCM) sensor signals of imprinted TiO_2_ nanoparticles (black) and MIP TiO_2_ sol-gel layer (grey) toward different concentrations of capric acid. The insert shows the corresponding frequency shifts when changing from fresh oil to used oil. Adapted from [52] with permission from Elsevier, 2007.

Dickert and coworkers [28,52,184] have designed molecularly imprinted titanate sol-gel materials and imprinted TiO_2_ nanoparticles for mass-sensitive detection of degraded products in used engine oils. Capric acid is chosen as the template for preparing the imprinted nanoparticles and sol-gel layers and the sensors are fabricated by spin-coating the sensitive layers on the surface of QCM electrodes [52]. Figure 8A shows the atomic force microscopy (AFM) image of imprinted TiO_2_ nanoparticles, and Figure 8B illustrates the sensor responses toward different concentration of capric acid as well as the frequency shift upon exposure to fresh and used oil samples. The imprinted QCM sensors exhibit significantly higher shifts in frequency for used oil sample due to the greater concentration of oxidized products in it. It is obvious that imprinted TiO_2_ nanoparticles are two-fold more sensitive to the oxidized oil products compared to imprinted sol-gel layers because of higher surface-to-volume ratio [52]. The results show that these sensors have good applicability and can be used in complex mixtures under highly corrosive environments. Furthermore, the imprinted sensors do not give false signals compared to other physical oil degradation sensing devices due to direct measurement of oxidized products in the degraded oil.

Mujahid et al. [185] have fabricated STW-based mass-sensitive devices with MIP TiO_2_ sol-gel material to monitor degradation products in automotive oil samples used for different time (hours) in an internal combustion engine. They also demonstrate excellent capability of the STW devices to work in complex mixtures. The advantage of using STW devices lies in their higher fundamental frequency compared to QCM, which leads to over 400% increase in the sensor responses. The STW sensor results are compared with FTIR measurements of used oil samples and show good correlation for the determination of oxidized products. Latif et al. [44] have further studied the applicability of imprinted TiO_2_ and SiO_2_ sol-gel layers in conductometric oil degradation sensors and have achieved promising results. Thus, MIP sol-gel layers and imprinted oxide nanoparticles can be integrated with both electronic and gravimetric devices to develop sensors for on-board assessment of automotive oil quality and quantitative determination of oxidized products in used engine oils.

### 3.5. Other Applications

There are many other applications of imprinted oxides and MIP/oxide nanohybrid materials including selective extraction and separation of biologically and environmentally important organic compounds and metal ions [186,187,188,189]; however, they are not part of this review since this review is dedicated only to the chemical sensing applications of imprinted oxides and MIP/oxide nanohybrids. In this section, we present the sensor applications that are not discussed in the previous sections: for instance, gas-phase sensing of organic vapors, detection of metal ions in aqueous medium, and recognition of cellular microorganisms.

#### 3.5.1. Gas-Phase Sensing of Organic Vapors

The molecular imprinting technology is not new for gas-phase detection of organic analytes [179]; however, it has seldom been applied to semiconducting oxide-based recognition elements. Albeit, it has been demonstrated that the inclusion of small metal nanoparticles in MIP-based receptors may actually collaborate to enhance their sensing properties [190,191], yet there are few examples of such chemical sensors compared to other recognition materials. Latif et al. [43] have applied imprinting procedure to fabricate TiO_2_ sol-gel layers on QCM for the detection of volatile organic compounds (VOC) and oil vapors. The sensitivity of imprinted TiO_2_ sol-gel is optimized by altering the Ti-alkoxide precursors: titanium tetrabutoxide (TBT), titanium tetrapropoxide (TPT), and titanium tetraethoxide (TEOT).

**Figure 9:**
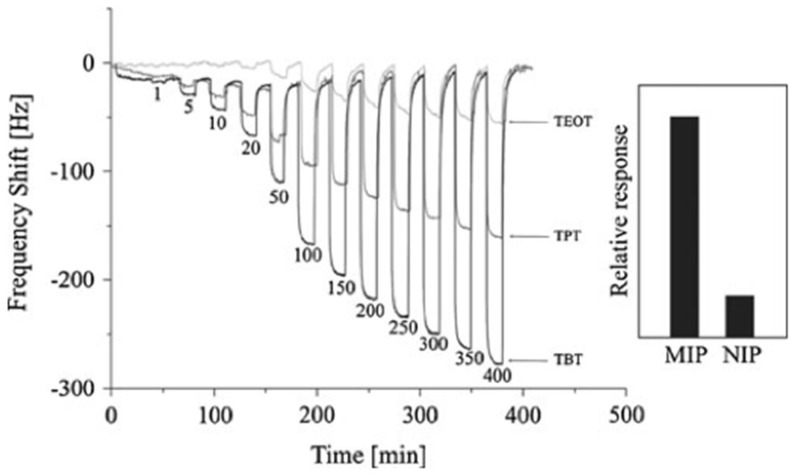
QCM sensor response of various molecularly imprinted TiO_2_ sol-gel layers produced from titanium tetrabutoxide (TBT), titanium tetrapropoxide (TPT), and titanium tetraethoxide (TEOT) precursors, exposed to 1–400 ppm of *n*-butanol vapors. Inset: Relative sensor responses of MIP (molecular imprinted polymer) and NIP (non-imprinted polymer) are also shown. Reproduced from [43] with permission from Springer, 2011.

The imprinted sensor with TBT precursor shows the highest sensitivity toward *n-*butanol, as shown in Figure 9. The QCM sensor can distinguish between the hydrocarbon isomers: *n-*octane and *iso*-octane, if TiO_2_ sol-gel is appropriately imprinted with the analyte of interest. Furthermore, the sensor is also applied to detect VOC in complex mixtures, i.e., degraded engine oil. Zhang et al. [192] have designed a chemiresistive sensor based on MIP/Ag-LaFeO_3_ nanoparticles for selective recognition of formaldehyde vapors. The MIP/Ag-LaFeO_3_ sensor exhibits promising sensor characteristics, e.g., high sensitivity for 0.5 ppm formaldehyde and fast response and recovery times (67 s and 104 s, respectively), compared to a simple Ag-LaFeO_3_ sensor reported earlier [193].

#### 3.5.2. Metal Ions Detection in Aqueous Medium

The ion imprinted polymers (IIP) are well known for their sensitivity and selectivity toward the targeted ions in aqueous solutions and real-time water and wastewater samples [194,195]. Roy et al. [196] have utilized an innovative approach to fabricate Eu^3+^-IIP on the rGO/silane-modified-Fe_3_O_4_ dendrite structure for electrochemical detection of Eu^3+^ ions. The square wave stripping voltammetry results demonstrate that the sensor is highly sensitive with a detection limit of 19 ng/L and selective toward Eu^3+^ ions. The sensor also exhibits ≥95.0% recoveries in real and spiked water samples.

**Figure 10:**
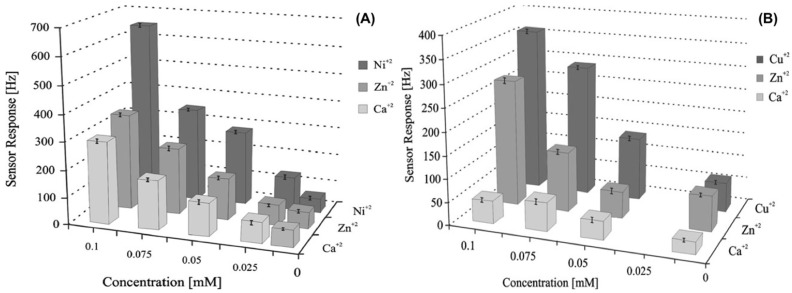
QCM sensor response of (**A**) Ni^2+^ and (**B**) Cu^2+^ ions imprinted SiO_2_ sol-gel modified with as amino-functional silane toward different metal ions (concentrations ranging from 0.01 to 0.1 mM). Adapted from [178] with permission from Springer, 2011.

Latif et al. [178] have developed a QCM based gravimetric sensors using Ni^2+^ and Cu^2+^ ions-imprinted SiO_2_ sol-gel modified with aminopropyltriethoxysilane. The QCM sensor shows significant selectivity and high sensitivity toward millimolar concentrations of targeted heavy metal ions, as shown in Figure 10. Authors state that the selectivity of ion imprinted sol-gel layers toward respective ionic template is not only governed by their ionic radii and imprinting effect, but also by the ligand field stabilization effect [178,197]. These results illustrate that imprinted oxide-based sol-gel materials have great potential to be used as selective recognition elements for the detection of metal ions.

#### 3.5.3. Detecting Cellular Microorganisms

**Figure 11:**
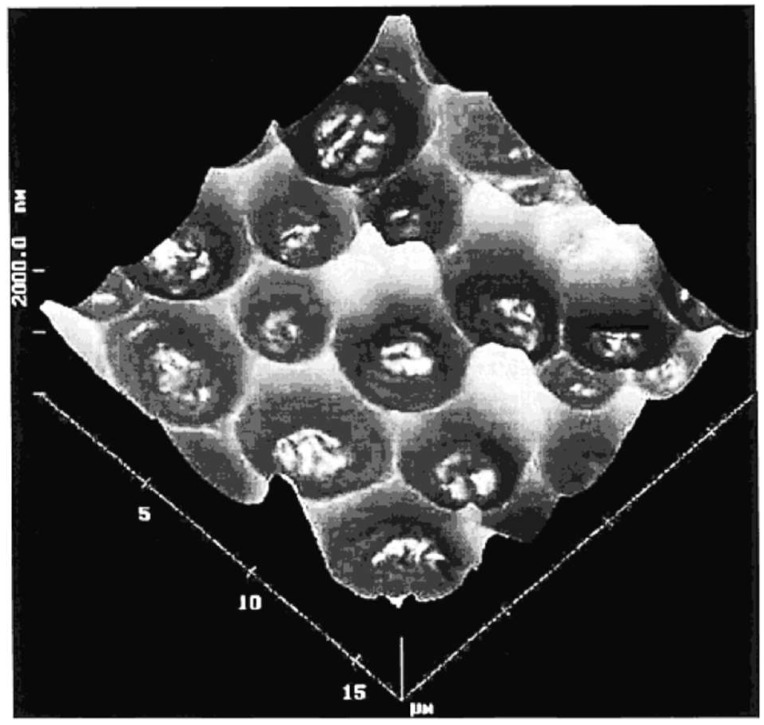
AFM images of a surface imprinted sol-gel layer based on titanium^IV^ ethylate imprinted with *S. cerevisiae* (yeast) cells. Reproduced from [26] with permission from the American Chemical Society, 2002.

Recognition of living cells and microorganisms such as bacteria is still a great challenge for advanced sensor technology and biomedical diagnostics [198,199,200]. Thus far, a technique that can reliably produce recognition elements for selective recognition of cells and microorganisms is surface imprinting via soft-lithography [201,202]. Dickert and Hayden have first presented the bioimprinting of sol-gel phases and their integration with QCM for the selective detection of yeast (*S. cerevisiae*) cells [26]. *S. cerevisiae* imprinted sol-gel phases are prepared by the acid-catalyzed hydrolysis of titanium^IV^ ethylate in the presence of poly(ethylene glycol) as plasticizer followed by a soft-lithographic stamping procedure. This process leads to the formation of highly robust scratch resistant coatings with cellular voids imprinted on their surface, as shown in Figure 11. The surface imprinted sol-gel sensor is capable of detecting *S. cerevisiae*; however, the sensor performance is not comparable to more sensitive polyurethane coatings [26].

Recently, Roy et al. [203] have developed an electrochemical sensor for the targeted detection of bacteria, *E. coli*. The sensor is fabricated by modifying GCE electrodes with bimetallic Ag-ZnO/GO nanocomposites followed by surface imprinting with *E. coli*. The proposed sensor can detect and capture 98% of the bacterial cells from the solution containing 10^5^ CFU/mL *E. coli*. These examples illustrate that it is quite possible to detect, capture and quantify cellular microorganisms with imprinted sol-gel and hybrid oxide-based recognition elements.

## 4. Summary and Outlook

A review of recent works on synthesis, fabrication, and applications of the selective recognition elements based on imprinted metal oxide sol-gel phases, nanostructured materials, and MIP/oxide nanohybrids is presented. From the examples discussed above, it is obvious that these imprinted oxide-based or oxide-containing MIP-based recognition elements can be easily combined with electrochemical, optical, and mass-sensitive or gravimetric devices for the selective detection of a variety of organic compounds, drugs, biomacromolecules, and cellular microorganisms. In majority of cases, these recognition elements present very high sensitivity and are therefore capable of detecting nanomolar-to-picomolar concentrations of various analytes. However, the primary requisites for real-life applications and commercialization of these sensors are device stability, specificity, and applicability in complex mixtures or real samples.

It is safely concluded that imprinted SiO_2_ and TiO_2_ sols can form highly stable, scratch-resistant sol-gel selective coatings that may work in harsh environments, e.g., at high temperatures and in highly corrosive mixtures, for longer operational time. They can effectively distinguish between structural isomers showing good specificity [43]. However, their rigid glassy nature may hinder diffusion of analytes into the deeper layers; thus, reducing their sensitivity or response time compared to MIP. It is suggested that these sensor characteristics may be improved by reducing the thickness of recognition layer or by fabricating mesoporous sol–gel coatings. Furthermore, these sol-gel phases can be bulk-imprinted or surface-imprinted depending on the analyte of interest and can be surface-functionalized using different functional silane monomers to optimize the sensor performance. Nevertheless, these sol-gel materials are certainly more stable than MIP.

The molecularly imprinted metal oxide nanoparticles present a great alternative for improving sensitivity compared to sol-gel phases because of their tiny size and high specific surface area availability for molecular interactions that is also demonstrated in a few studies discussed above [52]. They also reveal excellent stability and applicability in complex mixtures such as oxidized engine oils. These nanoparticles can also be imprinted, and surface modified to tailor their recognition properties. Although, metal oxides are known as efficient gas sensing elements and many devices based on metal oxide gas sensors are commercially available for a long time, we believe that a lot of work still needs to be done to accomplish their potential in other fields of chemical and biosensors via imprinting oxide nanomaterials.

Thanks to their excellent sensing properties, metal oxides can be incorporated in selective MIP coatings to enhance the overall sensor performance through a synergistic combination of MIP’s and oxides’ sensor characteristics. Thus, MIP/oxide nanohybrid-based recognition elements are amongst the best sensing materials encountered in this study. They are sensitive and show negligible to very low cross-sensitivity toward structural analogs of the target analyte. Moreover, these nanohybrid materials exhibit excellent applicability in real samples such as human blood serum, urine, river water, fruit juices, food samples etc. showing 100% recovery with low RSD [51,63,65,69,78,114,121]. Thus, MIP/oxide nanohybrid chemical sensors demonstrate remarkable performance.

Yet, many scientists are trying to develop more responsive recognition elements by combining different materials such as GO, CNT, β-CD, QD, etc. with MIP/oxide nanohybrids [81,113,117,157]; thus, producing multicomponent hybrid materials. The advantage of using multicomponent hybrid lies in better material properties: for example, integrating GO or CNT with MIP/oxide may increase conductivity and electrochemical activity thereby increasing the current response, while QD may improve the optical and photoelectrochemical activity thereby increasing the fluorescent sensor performance. Instead, β-CD may offer additional binding sites through host-guest inclusion chemistry thereby increasing sensitivity. In short, a multicomponent hybrid has higher sensitivity.

But, it is important to state that fabricating such multicomponent hybrid materials is a tedious task demanding long, multistep processes to obtain the final product, which may render their availability in commercial devices. Besides, one has to consider the essential compatibility between different components. It is difficult to accomplish sustained sensor performance without achieving good adhesion between different constituents of a multicomponent hybrid. Majority of the papers reviewed in this study do not focus on the reproducibility of these multicomponent hybrids over long working hours. Thus, it is hard to approve their long-term use in various sensor applications. In future, the research efforts may consider these perspectives besides expanding the use of different recognition elements to other relevant areas including point-of-care biomedical diagnostics, therapeutic drug assays, environmental monitoring, and industrial process control applications.

## Figures and Tables

**Table 1:** The electrochemical sensor characteristics of MIP/oxide nanohybrids for the detection of biologically relevant molecules.

Recognition Element	Analyte of Interest	Technique	Detection Limit (nM)	Linear Range (μM)	Matrix/Application	Ref.
MIP/MnO_2_-GO/CuO@Cu	Glucose	CV	53 × 10^3^	500–4400	Spiked water samples	[79]
MIP/SiO_2_@ITO	Resveratrol	CV/DPV	800	2–20	PBS	[78]
MIP/SnO_2_/MWNT@CE	Oleanolic acid	CV/LSV	0.019	1 × 10^−4^–4.38 × 10^−2^	*A. deliciosa* root sample	[80]
GO/SiO_2_-MIP@GCE	Dopamine	DPV	30	0.05–160	Human urine	[81]
MIP/ZnO@FTO	Dopamine	DPV	-	0.02–5	Human urine	[65]
MIP/CuO@GCE	Dopamine	CV	8	0.02–25	Human serum	[63]
MIP/ATO/SiO_2_@GCE	Norepinephrine	CV	33.3	0.1–15	Human blood serum	[82]
CuO/MIP@GCE	Tyrosine	CV/DPV	4	0.01–1	PBS	[64]
Fe_3_O_4_/GO/MIP@Au	Interleukin-8	CV/DPV	4 × 10^−5^	1 × 10^−7^–1 × 10^−5^	Saliva	[83]
MIP/ZnO@PGE	Calcitonin	CV/DPSV	3.09 (ng/L)	9.99 × 10^−3^–7.92 × 10^3^ (μg/L)	Human blood serum	[67]

MIP: Molecularly imprinted polymer, GO: Graphene oxide, r-GO: Reduced graphene oxide, ATO: antimony-doped tin oxide, MWNT: Multi-walled carbon nanotubes, CE: Carbon electrode, GCE: Glassy carbon electrode, PGE: Pencil graphite electrode, FTO: Fluorinated tin oxide, ITO: indium tin oxide, CV: Cyclic voltammetry, DPV: Differential pulse voltammetry, DPSV: Differential pulse stripping voltammetry, LSV: Linear sweep voltammetry, PBS: Phosphate-buffered saline solution.

**Table 2:** The sensor characteristics of MIP/oxide nanohybrids for the detection of harmful organic compounds.

Recognition Element	Analyte of Interest	Technique	Detection Limit (nM)	Linear Range (μM)	Matrix/Application	Ref.
MIP/SiO_2_-CdTe	Bisphenol A	Fluorescence	6	0.05–10	River water & milk	[113]
MIP/SnO_2_@ITO	Bisphenol A	PEC	1.2	2 × 10^−3^–0.5	Tap & river water	[114]
Ru(bpy)_3_^2+^/MWNT/nano-TiO_2_-nafion	Bisphenol A	ECL	4.1	0.01–2	River water	[115]
MIP/Au/TiO_2_@Au	4-Nonylphenol	CV	320	0.95–480	Pomfret & tap water	[116]
MIP/ZnO-MWNT-CS@ITO	*p*-Nitrophenol	CV/SWV	1	0.01–200	Spiked water samples	[117]
Fe_3_O_4_-MIP/rGO@GCE	17β-estradiol	CV/DPV	0.819	0.05–10	Water	[118]
MIP/Fe_3_O_4_-rGO@MGCE	Amaranth	DPV	50	0.05–50	Fruit drinks	[119]
MIP/Fe_3_O_4_-rGO/β-CD/IL/Au@GCE	Sunset yellow	DPV	2	0.005–2	Mirinda & minute maid	[74]
MIP/Fe_3_O_4_-GO	Vanillin	FI-CL	110	0.33–12	Vanilla drinks	[120]
MIP/Fe_3_O_4_	Mancozeb	CV	1.77	0.011–0.475	Vegetable & soil	[69]
MIP/TiO_2_@FTO	Chlorpyrifos	PEC	0.021	2.85 × 10^−5^–2.85 × 10^−1^	Spiked water samples	[121]
MIL-101(Cr)-MIP/Fe_3_O_4_-rGO/CS@GCE	Omethoate	CV/DPV	2.05 × 10^−5^	0.1–1 × 10^−7^	Cucumber & kidney bean	[122]
	Methamidophos		2.67 × 10^−4^	0.1–1 × 10^−6^		

FTO: Fluorine-doped tin oxide, MIL-101(Cr): Material of Institute Lavoisier (101; Cr), CS: Chitosan, β-CD: β-cyclodextrin, IL: Ionic liquid, MGCE: Magnetic glassy carbon electrode, PEC: Photoelectrochemical, ECL: Electrochemiluminescence, SWV: Square wave voltammetry, FI-CL: Flow injection chemiluminescence.

**Table 3:** The sensor characteristics of MIP/oxide nanohybrids for therapeutic drug monitoring.

Recognition element	Analyte of Interest	Technique	Detection Limit (nM)	Linear Range (μM)	Matrix/Application	Ref.
MIP sol-gel/ZnO	Tetracycline	Fluorescence	1270	2–120	Spiked water samples	[51]
MIP/Fe_3_O_4_/SiO_2_-MWNT-CS@CE	Benzylpenicillin	DPV	1.5	0.05–1000	Blood plasma	[154]
Imprinted MSSQ/TiO_2_@GCE	Procainamide hydrochloride	DPV	1.3	0.004–49.7	Human blood serum	[155]
Fe_3_O_4_/CdTe/MIP sol-gel	Ciprofloxacin	Fluorescence	392.3	0.15–1.81	Spiked urine samples	[58]
GO/MIP sol-gel@GCE	Paracetamol	DPV	20	0.1–80	Tablets & spiked urine	[50]
ATO/MIP sol-gel/CS@Pt	Clenbuterol	DPV	1.7	0.0056–6.3	Human serum	[156]
MIP sol-gel/Co./CS/β-CD/MWNT@ITO	Oxacillin	DPV	6.9	0.2–100	Human blood serum	[157]
MIP/MoS_2_-GN-CNT@GCE	Luteolin	LSV	9.0	0.04–2.0	Carrot & chrysanthemum tea samples	[158]

MSSQ: Methyl silsesquioxane, GN: Graphene, LSV: Linear sweep voltammetry.
